# SOCIAL WORK INVOLVEMENT IN ADVANCE CARE PLANNING POST 2015 MEDICARE POLICY CHANGE: A SYSTEMATIC REVIEW

**DOI:** 10.1093/geroni/igae098.2392

**Published:** 2024-12-31

**Authors:** Peiyuan Zhang, Yixuan Wang, John Cagle, Kaipeng Wang

**Affiliations:** University of Maryland Baltimore, Baltimore, Maryland, United States; Colorado State University, Fort Collins, Colorado, United States; University of Maryland Baltimore, Baltimore, Maryland, United States; University of Denver, Denver, Colorado, United States

## Abstract

Social workers have been identified as core members of the interdisciplinary team providing ACP but were excluded from the 2015 Medicare regulations reimburse clinicians for ACP discussions. This systematic review aims to synthesize evidence on social workers’ contributions to ACP since the 2015 Medicare Policy change, potentially informing policy reform to include them in reimbursement mechanisms. Using Preferred Reporting Items for Systematic Reviews and Meta-Analysis (PRISMA) guidelines, we searched PsycInfo, CINAHL, SocINDEX, and PubMed from January 2016 to July 2023. Peer-reviewed studies focusing on social worker involvement in ACP or advance directives (AD), with any study design except for case reports and reviews, were included. Studies with a mixed sample of healthcare professionals that did not compare social workers and other care professionals were excluded. Data were independently extracted by two investigators using pre-specified criteria. Quality was assessed using Critical Appraisal Skills Program (CASP) tools and STORBE checklists. Key themes include social workers’ (1) knowledge and competency of ACP, (2) attitudes and perceptions of ACP, (3) involvement in ACP practices, (4) perceived roles and responsibilities, and (5) effects of social work interventions on ACP engagement. Findings highlight social workers’ positive attitudes towards ACP and perceived responsibility in ACP engagement, which underscore social workers’ prominent role in educating ADs and facilitating ACP conversations. This review provides the most recent synthesis of relevant findings about social work ACP practices after 2015 Medicare reimbursement for ACP, which has important policy implications in including social workers in the ACP reimbursement mechanism.

